# A novel, single-amplification PCR targeting mitochondrial genome highly sensitive and specific in diagnosing malaria among returned travellers in Bergen, Norway

**DOI:** 10.1186/1475-2875-12-26

**Published:** 2013-01-22

**Authors:** Christel G Haanshuus, Stein C Mohn, Kristine Mørch, Nina Langeland, Bjørn Blomberg, Kurt Hanevik

**Affiliations:** 1 National Centre for Tropical Infectious Diseases, Department of Medicine, Haukeland University Hospital, Bergen, Norway; 2 Institute of Medicine, University of Bergen, Bergen, Norway

**Keywords:** Malaria, Diagnostics, PCR, Amplification, Sequencing, Mitochondrial DNA, 18S, Sensitivity, Gametocytes, Returned travellers

## Abstract

**Background:**

Nested PCR is a commonly used technique in diagnosis of malaria owing to its high sensitivity and specificity. However, it is time-consuming, open to considerable risk of contamination and has low cost-efficiency. Using amplification targets presented in multiple copies, such as rRNA 18S, or mitochondrial targets with an even higher copy number, might increase sensitivity.

**Methods:**

The sensitivity and specificity of two newly designed *Plasmodium* genus-specific single-round amplification PCR programmes, based on previously published primers targeting 18S and mitochondrial genome, were compared with a widely used nested 18S PCR. Analyses of dilution series from *Plasmodium falciparum* reference material were performed, as well as retrospective analyses of 135 blood samples, evaluated by routine microscopy, from 132 fever patients with potential imported malaria. Sequencing of the 220 bp mitochondrial PCR products was performed.

**Results:**

At the threshold dilution 0.5 parasites/μl, the sensitivity of the mitochondrial PCR was 97% (29/30 parallels), that of the single-round 18S PCR 93% and the reference nested 18S PCR 87%. All three assays detected as low as 0.05 p/μl, though not consistently. In the patient cohort, malaria was diagnosed in 21% (28/135) samples, defined as positive by at least two methods. Both single-round amplification assays identified all malaria positives diagnosed by nested PCR that had sensitivity of 96% (27/28). The mitochondrial PCR detected one additional sample, also positive by microscopy, and was the only method with 100% sensitivity (28/28). The sensitivity and specificity of the mitochondrial PCR were statistically non-inferior to that of the reference nested PCR. Microscopy missed two infections detected by all PCR assays. Sequencing of the genus-specific mitochondrial PCR products revealed different single nucleotide polymorphisms which allowed species identification of the 28 sequences with following distribution; 20 *P. falciparum*, six *Plasmodium vivax*, one *Plasmodium ovale* and one *Plasmodium malariae*.

**Conclusions:**

In this study, design of PCR programmes with suitable parameters and optimization resulted in simpler and faster single-round amplification assays. Both sensitivity and specificity of the novel mitochondrial PCR was 100% and proved non-inferior to that of the reference nested PCR. Sequencing of genus-specific mitochondrial PCR products could be used for species determination.

## Background

More than three billion people world-wide are exposed to malaria, resulting in an estimated 200 million malaria cases and 1.2 million deaths in 2010 [1]. Routine diagnosis is usually done by microscopy and/or rapid diagnostic antigen detection tests, and may be influenced by factors such as the technologists’ level of experience, and equipment quality [2,3]. Several molecular techniques have been developed, including polymerase chain reaction (PCR), with the aim of increasing the sensitivity and specificity [4,5].

Malaria PCR was first introduced in 1990 [6], and subsequently has evolved with new amplification methods such as Real-Time PCR and loop-mediated isothermal amplification (LAMP) [7,8]. Both the latter methods have advantages over conventional PCR in turnaround time and in practical use as the detection step is incorporated into the amplification step. Nevertheless, the nested PCR originally described by Snounou *et al.* in 1993 [9] and improved in 1999 [10], remains commonly used and often regarded as a gold standard/reference method. Nested PCR exhibits high sensitivity and specificity due to two amplification steps, but is time-consuming, open to considerable risk of contamination and has low cost-efficiency [11-16].

High sensitivity has also been achieved by using amplification targets existing in multiple copies in the *Plasmodium* genome [17]. A common target is the conserved small subunit ribosomal RNA 18S locus [9,18,19] which in the *Plasmodium falciparum* chromosomal genome exists in five to eight copies depending on the strain [20]. Snounou *et al.*[9] reported a sensitivity of one to 10 parasites per microlitre (p/μl) of blood using nested PCR with 18S as the target gene. Polley *et al.*[21] reported a sensitivity of 5 p/μl introducing a LAMP method using an amplification target on the 6 kb mitochondrial genome. In comparison, a LAMP assay employing primers targeting 18S had a sensitivity limited to approximately 100 copies of the gene for *P. falciparum*[19]. Early ring stage *P. falciparum* parasites typically have one mitochondrial organelle, which contains about 20 copies of the 6 kb genome, while mature gametocytes have as much as four to eight mitochondrial organelles [22,23]. Although one would expect assays using mitochondrial targets to show higher sensitivity given the higher copy number, PCR-based methods targeting 18S are commonly the methods of choice [17,24].

The main aim of this study was to design practical single-round amplification *Plasmodium* genus-specific PCR assays, based on previously described primers targeting the 18S locus [9] and the mitochondrial genome [21], with sensitivity and specificity non-inferior to nested PCR [10]. Comparisons were performed using reference material and samples from a cohort of fever patients with potential imported malaria in Bergen, Norway.

## Methods

### Patient materials, positive controls and reference sample

The patient material used in this study had been collected between 2006 and 2011 at Haukeland University Hospital, Bergen, Norway. It included 135 whole blood samples from a cohort of 132 fever patients with potential imported primary or recurrent malaria. As part of the routine work-up these samples had been previously analysed for malaria parasites on Giemsa-stained, thin and thick slides by experienced microscopists. The routine microscopy results and clinical information were collected retrospectively from patient files.

External DNA controls extracted from *P. falciparum*, *Plasmodium vivax*, *Plasmodium ovale* and *Plasmodium malariae* supplied by the Centre for Tropical Diseases, McGill University (Quebec, Canada) [25] were used in the validation of the genus-/ and species-specific PCR assays in the study. In addition, an external reference sample of *P. falciparum*, US 04 F Nigeria XII (World Health Organization, Geneva, Switzerland), was used to examine the sensitivity of the genus-specific PCR assays. *P. falciparum* was the only cultivated species accessible. The reference material contained exclusively ring stage parasites in a concentration of 200 p/μl. Employing template from the reference material together with extracted DNA from blood of a Norwegian malaria negative volunteer, a combination of two 10-folds dilutions series were prepared giving the following series: 10 p/μl, 5 p/μl, 1 p/μl, 0.5 p/μl, 0.1 p/μl, 0.05 p/μl, and 0.001 p/μl.

From all blood samples DNA was extracted using QIAamp DNA Blood Mini Kit (Qiagen, Hilden, Germany) according to the manufacturer’s instructions. Both blood and extracted DNA material was stored at -20°C prior to application.

## PCR methods

Three PCR assays, two genus-specific and one species-specific, were assessed in the cohort of patient samples described above. The genus-/ and species-specific nested 18S PCR as described by Singh *et al.*[10] was included as a reference method. In each PCR assay the reaction mixtures contained 2 μl of DNA template and 12.5 μl 2X HotStarTaq Master Mix (Qiagen) at a total volume of 25 μl. The amplifications were performed by using GeneAmp PCR System 9700 (Applied Biosystems, Carlsbad, CA, USA), and the PCR products were analysed by electrophoresis using 2% SeaKem® agarose gel (Lonza, Rockland, ME, USA) with 1X GelRed™ (Biotium, Hayward, CA, USA). Concentrations of primers and additional MgCl_2_ were optimized for each assay examined as described below.

One genus-specific PCR assay employed primers rPLU 6 and rPLU 5 (Table 1) targeting 18S. The amplification conditions were modified and optimized from the original nested [9] to a single-round amplification assay with cycle parameters as follows: step 1, 95°C for 15 min; step 2, denaturation at 95°C for 10 sec; step 3, annealing at 63°C for 10 sec; step 4, extension at 72°C for 75 sec; steps 2-4 repeated 50 times; and step 5, 72°C for 10 min.

**Table 1:** Primers applied for the amplifications and sequencing examined in this study

**Primer**^**1**^	**Sequence**	**Published by**
**rPLU 6 forward**	5^′^-tta aaa ttg cag tta aaa cg	Snounou *et al. *[9]
**rPLU 5 reverse**	5^′^-cct gtt gtt gcc tta aac ttc	Snounou *et al. *[9]
**PgMt19 F3 forward**	5^′^-tcg ctt cta acg gtg aac	Polley *et al. *[21]
**PgMt19 B3 reverse**	5^′^-aat tga tag tat cag cta tcc ata g	Polley *et al. *[21]
***Plasmodium falciparum *forward**	5^′^-aac aga cgg gta gtc atg att gag	Padley *et al. *[26]
***Plasmodium vivax *forward**	5^′^-gag cgt tca aag caa aca ga	This study
***Plasmodium ovale *forward**	5^′^-ctg ttc ttt gca ttc ctt atg c	Padley *et al. *[26]
***Plasmodium malariae *forward**	5^′^-cgt taa gaa taa acg cca agc g	Padley *et al. *[26]
**Species-specific reverse**	5^′^-gta tct gat cgt ctt cac tcc c	Padley *et al. *[26]

^1^ The primers were obtained from Eurogentec (Seraing, Belgium).

The other genus-specific assay was a new single-round amplification PCR using primers PgMt19 F3 and PgMt19 B3 (Table 1), targeting the mitochondrial genome and previously employed in a LAMP assay [21]. The primers were analysed using Oligo v6 primer analysis software (Molecular Biology Insights, Cascade, CO, USA), and tested with Basic Local Alignment Search Tool (BLAST, National Center for Biotechnology Information, Bethesda, MD, USA) before being considered suitable for use in a conventional PCR assay. Subsequently, the following amplification conditions were designed: step 1, 95°C for 15 min; step 2, denaturation at 95°C for 10 sec; step 3, annealing at 62°C for 10 sec; step 4, extension at 72°C for 15 sec; steps 2-4 repeated 50 times; and step 5, 72°C for 10 min. Reaction mixture for both assays contained 250 nM of each primer, and additionally 4 mM MgCl_2_ (New England BioLabs, Ipswich, MA, USA).

The *P. falciparum*, *P. vivax*, *P. ovale* and *P. malariae* species-specific PCR protocol was employed on all genus-specific PCR positive samples applying primers targeting 18S previously published by Padley *et al.*[26]. As opposed to the original multiplex assay, each sample was analyzed in four separate reaction mixtures to avoid difficulties in species interpretation due to similar product sizes. Only a few single nucleotide polymorphisms (SNPs) distinguish the different forward primers’ hybridization sites. In order to avoid non-specific cross-binding between *P. falciparum* and *P. vivax* samples, a new *P. vivax* forward primer (Table 1) was designed using Oligo v6, the sequence alignment editor software BioEdit v7 (Tom Hall, Carlsbad, CA, USA) and BLAST. The amplification conditions were modified and amplification time substantially reduced from six to two hours. The new cycling parameters were: step 1, 95°C for 15 min; step 2, denaturation at 95°C for 10 sec; step 3, annealing at 65°C for 10 sec; step 4, extension at 72°C for 30 sec; steps 2-4 repeated 45 times; and step 5, 72°C for 10 min. The reaction mixtures contained additional MgCl_2 _which was optimized as follows: 4 mM for *P. ovale*, 2 mM for *P. falciparum* and *P. malariae*, and 1 mM for *P. vivax*. The primer concentrations were 250 nM with an exception of 150 nM for *P. ovale.*

### Sequencing

For quality assurance purposes, all PCR products from genus-specific positive samples using primers PgMt19 F3&B3 (Table 1) were sequenced in both directions. The PCR products were purified with ExoSAP-IT® (USB Corporation, Cleveland, OH, USA) according to the manufacturer’s instructions, prior to the following cycle conditions being applied using GeneAmp PCR System 9700 (Applied Biosystems): step 1, 96°C for 10 sec; step 2, 62°C for 5 sec; step 3, 60°C for 4 min; steps 1-3 repeated 27 times. Each reaction mixture contained 1 μl BigDye v1.1 (Applied Biosystems), 2 μl sequencing buffer 5X (Applied Biosystems), 0.5 μM primer, and 1 μl template at a total volume of 10 μl. The sequences were obtained with the ABI PRISM® 3730 DNA Analyzer (Applied Biosystems). BioEdit v7 was used prior to sequence identification using BLAST.

### Statistical methods

Proportions were compared applying prtest command using Stata 11 (Stata Corp, College Station, Texas, USA), and differences in sensitivity and specificity with 95% confidence intervals (95% CI) were calculated. Non-inferior of the test was considered proved if the upper boundary of the 95% CI of the sensitivity difference was less than a predefined delta of 5%.

### Ethics

The study was approved by the Regional Committee for Ethics in Medical Research (No.2011/942).

## Results

**Figure 1:**
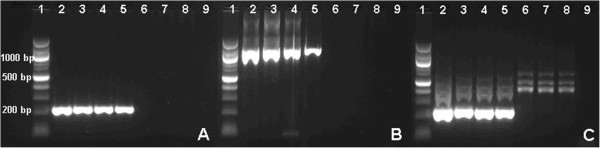
**Sensitivity of three different *Plasmodium *genus PCR protocols.** A dilution series, run in 30 parallels, was prepared from a 200 p/μl stock of *Plasmodium falciparum*, US 04 F Nigeria XII. The 2% agarose gel picture shows a typical parallel for each assay; new single-round amplification mitochondrial PCR employing primers PgMt19 F3 & B3 [21 ](**A**), modified single-round amplification 18S PCR employing primers rPLU 6 & 5 [9 ](**B**), and gold standard genus-specific nested 18S PCR [10 ](**C**). The product sizes are 220 base pair (bp), 1200 bp, and 250 bp, respectively. Lane 1 = 100 bp DNA Ladder (New England BioLabs), lane 2 = 10 p/μl, 3 = 5 p/μl, 4 = 1 p/μl, 5 = 0.5 p/μl, 6 = 0.1 p/μl, 7 = 0.05 p/μl, 8 = 0.001 p/μl, and 9 = no template. The new mitochondrial PCR had more defined bands than the modified 18S PCR, and especially the nested 18S PCR.

The sensitivity of detection was examined for the three genus-specific amplification assays; the new single-round amplification mitochondrial PCR employing primers PgMt19 F3&B3 [21], the modified single-round amplification 18S PCR employing primers rPLU 6&5 [9], and nested 18S PCR [10 ]as the reference method. Based on 30 parallels of the described dilution series, the 0.5 p/μl dilution proved to be the threshold detection level for all three assays (Figure 1). The mitochondrial PCR detected 0.5 p/μl with 97% sensitivity (29/30), while the modified 18S PCR and nested 18S PCR detected 0.5 p/μl with 93% (28/30) and 87% (26/30) sensitivity, respectively. At the threshold detection level of 0.5 p/μl, the sensitivity of the mitochondrial PCR, was statistically non-inferior to that of the reference nested 18S PCR, as the upper boundary of the 95% CI (-23.8% to 3.8%) of the sensitivity difference (10.0%) was less than the predefined delta of 5%. Although, the sensitivity of the modified 18S PCR also was higher than that of the reference nested 18S PCR in absolute numbers, statistical non-inferiority could not be proved in this sample size (sensitivity difference -6.7%, 95% CI -21.8% to 8.4%). All three assays detected positive DNA as low as 0.05 p/μl, though not consistently so by any method.

After having determined sensitivity of detection in standardized material, the 135 patient samples (132 patients) were screened for the presence of malaria by the three genus-specific PCR assays. Among these samples, 21% (28/135) were defined as malaria positive by at least two of the methods among microscopy and the three different genus-specific PCR assays. The new mitochondrial PCR was 100% sensitive detecting all 28 positives. Both the modified 18S PCR and the nested 18S PCR detected 27 of the positives corresponding to a sensitivity of 96%. Routine microscopy detected 26 of the positives corresponding to a sensitivity of 93% (Table 2). The mitochondrial PCR detected one positive sample not detected by any of the two 18S PCR assays, which was also positive by microscopy. Two positive samples not detected by microscopy were detected by all three PCR assays (Table 2). The sensitivity of the mitochondrial PCR was statistically non-inferior to that of the reference 18S PCR, as the upper boundary of the 95% CI (-10.4% to 3.3%) of the sensitivity difference (-3.6%) was less than the predefined delta of 5%. Although the sensitivity of the modified 18S PCR was equal to that of the reference nested 18S PCR in absolute numbers, statistical non-inferiority could not be proved in this patient cohort (sensitivity difference 0%, 95% CI -9.7% to 9.7%).

**Table 2:** Genus-specific results from a cohort of 132 fever patients with potential imported malaria

**Samples (n = 135)**	**New single-round amplification mitochondrial (PCR*PgMt19 F3 & B3 ***[21]**)**	**Modified single-round amplification 18S PCR *(rPLU 6 & 5 ***[9]**)**	**Genus-specific nested 18S PCR **[10]	**Microscopy**
**Positive**	28	27	27	26
**Negative**	107	108	108	109

None of the malaria-negative patients had false positive tests by any of the diagnostic methods, thus all the tests had 100% specificity in this cohort. The specificity of both the mitochondrial PCR and the modified 18S PCR were statistically non-inferior to that of the reference nested 18S PCR (specificity difference 0.0%, 95% CI 0.0 to 0.0%).

**Figure 2:**
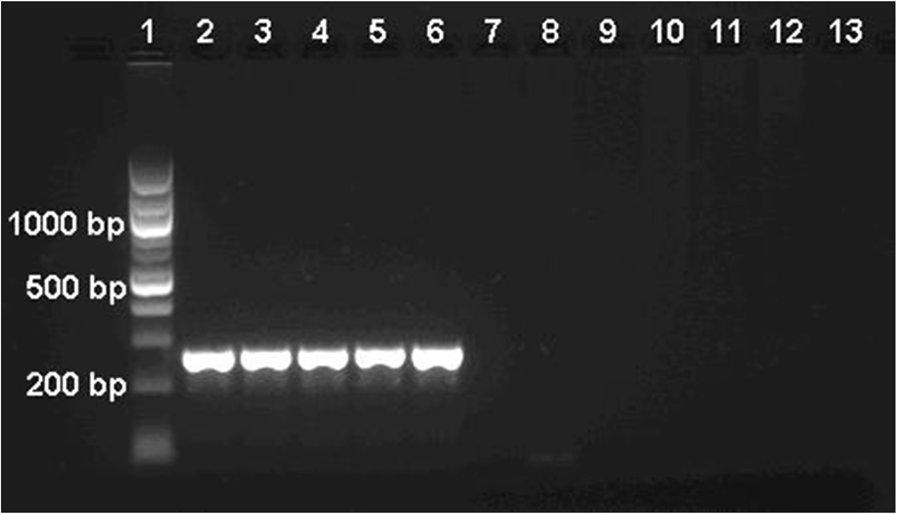
**No cross-binding reactions applying new *Plasmodium vivax *forward primer in modified species-specific 18S PCR **[26]**.** The original *Plasmodium vivax* primer from a multiplex PCR [26] cross-bonded with *Plasmodium falciparum* when applied in the modified species-specific 18S PCR [26]. The new *Plasmodium vivax* (Pv) primer was cross tested against different *Plasmodium falciparum* (Pf) patient samples with diverse levels of high parasitaemia. The results showed on a 2% agarose gel. Lane 1 = 100 bp DNA Ladder, lanes 2-6 = Five different positive Pv patient samples, lane 7 = 2% Pf, lane 8 = 2-3% Pf, lane 9 = 7% Pf, lane 10 = 7-10% Pf, lane 11 = 10-15% Pf, lane 12 = 20% Pf, and lane 13 = No template. The product size of Pv is 241 bp.

The 28 malaria positive samples were all further examined by two species-specific 18S amplification assays; an assay modified from a multiplex PCR [26], and species-specific nested PCR assay [10] as the reference method. The first assay, which was modified from a multiplex into four separate reactions with a new *P. vivax* forward primer, as described above, showed no cross-bindings between the new *P. vivax* primer and *P. falciparum* positive samples (Figure 2). Previously observations showed that the original *P. vivax* forward primer unspecifically cross-bonded with *P. falciparum* positive samples, especially when the parasitaemia was high.

**Figure 3:**
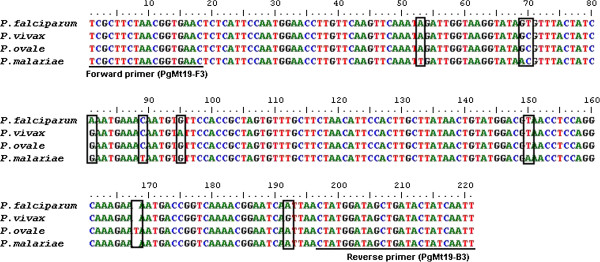
**Sequencing of *Plasmodium *genus-specific mitochondrial PCR products reveals polymorphisms consequently identifying malaria species.** Sequencing of the genus-specific mitochondrial PCR products, employing primers PgMt19 F3&B3 [21], gave high-quality sequences of full length. Run through BLAST the results showed that the four species were distinguished by two to six single nucleotide polymorphisms and one insert/deletion consequently allowing sequencing to be used as an alternative species-specific determination method in our cohort.

Sequencing of the genus-specific mitochondrial PCR products was performed giving high-quality sequences of full length. When the sequences were run through BLAST the results showed two to six SNPs and one insert/deletion which allowed species determination in all the 28 sequences, revealing 20 *P. falciparum*, six *P. vivax*, one *P. ovale*, and one *P. malariae* sequences (Figure 3, Table 3). Both species-specific 18S PCR assays identified 18 *P. falciparum*, six *P. vivax*, one *P. ovale*, and one *P. malariae* single infections, and one double infection of *P. falciparum* and *P. malariae* (Table 3). While the routine microscopy had identified 20 *P. falciparum*, three *P.m vivax*, and one *Plasmodium knowlesi*/inconclusive single infections, and two samples with *P. falciparum* in double infection with *P. ovale* and *P. vivax*, respectively (Table 3).

**Table 3:** Species-specific results among malaria genus positive samples

**Samples (n = 28)**	**Sequencing *PgMt19 F3 & B3 ***[21]	**Modified species-specific 18S PCR **[26]	**Species-specific nested 18S PCR **[10]	**Microscopy**
18	Pf	Pf	Pf	Pf
1	Pf	Pf + Pm	Pf + Pm	Pf
1	Pf	Negative	Negative	Pf
3	Pv	Pv	Pv	Pv
1	Pv	Pv	Pv	Negative
1	Pv	Pv	Pv	Pf + Pv
1	Pv	Pv^1^	Pv^1^	Pk^2^
1	Po	Po	Po	Pf + Po
1	Pm	Pm	Pm	Negative

**Abbreviations:** Pf, *Plasmodium falciparum*; Pv, *Plasmodium vivax*; Po, *Plasmodium ovale*; Pm, *Plasmodium malariae*; Pk, *Plasmodium knowlesi.*

^1^*Plasmodium knowlesi* primers were not included in the assay.

^2^ Evaluated as inconclusive species by microscopy, but with emphasis on possible *Plasmodium knowlesi* infection.

In seven samples (six patients) the different methods had discordant results (Table 4). Based on the combined results from routine microscopy, all PCR assays and sequencing, these samples were evaluated giving the following interpretations; sequencing as a diagnostic method missed *Plasmodium malariae* in a double infection of *P. falciparum* and *P. malariae*, all 18S PCR assays (genus-/and species-specific) missed one *P. falciparum* positive sample detected by both the new mitochondrial PCR and microscopy, and routine microscopy missed one *P. malariae* and one *P. vivax* single infection as well as *P. malariae* in a double infection of *P. falciparum* and *P. malariae*. Microscopy incorrectly evaluated two single infections as double infections with *P. falciparum*, and incorrectly evaluated a *P. vivax* as *P. knowlesi* infection (Table 4).

**Table 4:** Seven samples (six patients) with discordant results between the different methods

**Patient**	**Microscopy**	**Parasitaemia by microscopy**	**18S PCR assays**	**Mitochondrial PCR/sequencing**	**Primary/recurrent infection**	**Place of infection**	**Interpretation**
**P 1**	Negative	0^1^	Pm	Pm	Primary	Ghana	Pm overlooked by microscopy
**P 2**	Negative^2^	0^1^	Pv	Pv	Primary	New Guinea	Pv overlooked by microscopy
**P 2**	Pf, Pv or Pk	1%	Pv	Pv	Recurrent (Relapse)	New Guinea	Inconclusive/ incorrect diagnosis by microscopy
**P 3**	Pf + Po	<1%	Po	Po	Recurrent (Relapse)	Uganda	Over-diagnosed mixed infection by microscopy
**P 4**	Pf	<1%	Pf + Pm	Pf	Primary	Liberia	Under-diagnosed mixed infection by microscopy/sequencing
**P 5**	Pf + Pv	1%	Pv	Pv	Primary^3^	SEA or CA	Over-diagnosed mixed infection by microscopy
**P 6**	Pf ^4^	<1%	Negative	Pf	Recurrent (Recrudescence)	Guinea	Un-detected low Pf parasitaemia by 18S PCR

**Abbreviations:** Pf, *Plasmodium falciparum*; Pv, *Plasmodium vivax*; Po, *Plasmodium ovale*; Pm, *Plasmodium malariae*; Pk, *Plasmodium knowlesi*; SEA, South east Asia; CA, Central America.

^1^ Not detected any parasites.

^2^ Positive microscopy two days later (1% parasitaemia) and then the patient was diagnosed with severe malaria and treatment initiated.

^3^ Not given hypnozoite-eradicating treatment and re-admitted four weeks later with relapse of *Plasmodium vivax.*

^4^ Only one parasite detected.

## Discussion

The two *Plasmodium* genus-specific single-round amplification PCR assays presented in this study were as follows: a new mitochondrial PCR employing primers PgMt19 F3&B3 [21], and a modified 18S PCR employing primers rPLU 6&5 [9]. By designing single-round amplification assays, the cost-efficiency, turnaround time, contamination risk, and the possibility of technical errors are considerably reduced compared with the nested PCR reference method [10]. Previously published 18S primers, rPLU3&4 [10], were also considered for single-round amplification because of short product size (250 bp *versus* 1200 bp), but discarded due to high annealing temperatures, 71 and 75°C (contra 63 and 64°C for rPLU6&5). Snounou and Singh [9,10] reported that the 18S primers showed reduced sensitivity in single-round PCR amplifications, however this study shows that single-round amplifications have the same sensitivity and specificity as nested PCR given suitable parameters and optimisation. The new PCR programs were designed with a high number of cycles and high concentration of additional magnesium chloride, so that the sensitivity of nested PCR was maintained. Furthermore, due to the quick cycles in the designed programs and the high annealing temperatures, also the specificity of nested PCR was maintained despite the high number of cycles. The results from the dilution series showed that both the single-round amplification assays detected a higher number of parallels with positive DNA at the threshold dilution level of 0.5 p/μl compared to the nested PCR reference method. In the patient material the two single-round assays correctly identified all the positives and negatives found by the nested PCR, with the exception of one sample positive only by mitochondrial PCR and microscopy. Statistically the sensitivity of the mitochondrial PCR was non-inferior to that of the reference nested 18S PCR in this patient cohort as well as in the dilution series (at the threshold 0.5p/ μl dilution). Although the sensitivity of the modified 18S PCR could not be proven to be statistically non-inferior to nested PCR in this limited sample size, the test was equal to or better than the nested PCR in absolute numbers. The specificities of both single-round amplification assays were statistically non-inferior to that of the nested PCR.

In *Plasmodium* parasites the mitochondrial genome is presented in a higher copy number than 18S, especially in gametocytes. While the copy number of 18S locus varies depending on strains, and not on the stages of the parasite since it is located on the chromosomal genome [20], the multiple mitochondrial genome is located in the mitochondrial organelles which can varies in number through the different parasite’s stages [22,23]. The results from the dilution series, containing ring stage parasites exclusively, showed the same threshold detection level for all three genus-specific PCR assays, though the mitochondrial PCR had highest sensitivity on this detection level. In ring stage parasites the copy number of the mitochondrial genome is approximately three to four times higher than the copy number of the 18S locus. Compared to 18S PCR, the mitochondrial PCR detected one more sample among the patient material, and notably none of the four 18S PCR assays (two genus-/ and two species-specific) detected this sample. The patient had recrudescence following primary *P. falciparum* infection two weeks earlier. Maturation of *P. falciparum* gametocytes takes eight to 10 days [27,28]. Although microscopy detected one single ring stage parasite, the difference in sensitivity in this sample is possibly due to submicroscopic gametocytaemia; the copy number of the mitochondrial genome in a gametocyte is approximately 10 to 32 times higher than the copy number of the 18S locus.

Due to short product size, 220 bp, the amplification time with the new mitochondrial PCR programme takes one-and-a-half hours less than the modified 18S PCR programme, and three hours less than nested 18S PCR [10]. Another advantage of the short product size, was that the mitochondrial PCR products could be easily full-length sequenced. As an alternative to species-specific PCR, the SNPs found in the sequences allowed for specific species determination (Figure 3). However the validity of the SNPs warrants further investigation to evaluate if the SNPs are universal in different strains of the species. All of the 28 sequences correctly identified species compared to the species confirmed by either the species-specific 18S PCR assays or microscopy. However, sequencing only identified *P. falciparum* species in a double infection with *P. malariae*, which was detected by both the species-specific 18S PCR assays. This can be explained by *P. falciparum* predominance due to its ability to induce high parasitaemia. Despite missing the less numerous species in mixed infections, sequencing of the mitochondrial PCR products can be useful in species diagnosis. Compared to a species-specific multiplex PCR, the sequencing method avoids unwanted cross-bindings due to similar primers’ hybridization sites, difficulties in interpreting results due to similar product sizes, and lower sensitivity due to competing primers. Due to high quality sequences this method only requires one reaction for every positive sample to perform species determination, compared to four/five in non-multiplex species-specific PCR assays. Sequencing of positive mitochondrial PCR products also allows for identification of species where species-specific 18S PCR assays may fail to detect low parasitaemia, as shown in the sample with recrudescence in this patient cohort. The simple and rapid mitochondrial PCR assay, with the advantages of high sensitivity and that species-specific sequencing is possible in positive samples, might therefore have a value in screening purposes, especially in large scale epidemiologic and surveillance studies as well as in diagnostics.

## Conclusions

In this study two simple and rapid single-round amplification assays for detection of malaria were described. The novel mitochondrial PCR was the only method with 100% sensitivity in this patient cohort, and both its sensitivity and specificity was statistically non-inferior to that of the reference 18S nested PCR [10]. The method may be of particular value in samples of low parasitaemia/gametocytaemia and in large-scale studies. Sequencing of the genus-specific mitochondrial PCR products or developing the assay to real-time PCR could be an alternative to species-specific PCR in species determination.

## Competing interests

The authors declare that they have no competing interests.

## Authors’ contributions

CGH designed, developed and optimized the PCR programs, performed the analyses, and wrote the first draft of the manuscript. SCM contributed in the development of the PCR methods. KM contributed in providing the clinical data, and in the clinical interpretations. NL initiated the collection of the patient material. BB performed the statistical analysis. KH was involved in including the reference material. All authors contributed to planning of the study, interpretation of the results, revision of the manuscript, and approved the final version.
